# Clinical Utility and Accuracy of Point-of-Care Testing for Anti-TNF Drug Monitoring and Loss of Response

**DOI:** 10.1093/ibd/izaf101

**Published:** 2025-05-07

**Authors:** Christoph Teichert, Suzanne I Anjie, Toer W Stevens, Bayda Bahur, Kurtis R Bray, Krisztina B Gecse, Geert R D’Haens

**Affiliations:** Department of Gastroenterology and Hepatology, Amsterdam University Medical Centre, Amsterdam, The Netherlands; Department of Gastroenterology and Hepatology, Amsterdam University Medical Centre, Amsterdam, The Netherlands; Department of Gastroenterology and Hepatology, Amsterdam University Medical Centre, Amsterdam, The Netherlands; ProciseDx Inc., 9449 Carroll Park Drive, San Diego, CA, USA; ProciseDx Inc., 9449 Carroll Park Drive, San Diego, CA, USA; Department of Gastroenterology and Hepatology, Amsterdam University Medical Centre, Amsterdam, The Netherlands; Department of Gastroenterology and Hepatology, Amsterdam University Medical Centre, Amsterdam, The Netherlands

**Keywords:** therapeutic drug monitoring, anti-TNF, point-of-care testing, loss of response

## Abstract

**Background and Aims:**

Point-of-care tests (POCT) enable immediate measurement of anti-TNF blood concentrations. This study examined the association between loss of response (LOR) to infliximab (IFX) or adalimumab (ADL) and serum concentrations measured with POCT and enzyme-linked immunosorbent assay (ELISA) in inflammatory bowel disease (IBD) patients.

**Methods:**

Patients with IBD with stored IFX or ADL serum samples were recruited. POCT was conducted, agreement with ELISA was evaluated using Bland–Altman plots. The primary endpoint was LOR defined as change in therapy, IBD-related surgery, new actively draining fistula, and/or endoscopic deterioration. ROC curves and quartile analysis assessed the association between concentrations and LOR.

**Results:**

A total of 176 patients were included (92 IFX/84 ADL, 154 Crohn’s disease, and 22 ulcerative colitis). Median follow-up time was 20 months (interquartile range 9-38). LOR occurred in 37/84 (44%) ADL users and 55/92 (60%) IFX users. Median serum concentrations were significantly lower in LOR patients compared with sustained response, measured by both techniques for ADL (POCT: 6.45 vs 13.48 µg/mL, *P* <.001; ELISA: 4.80 vs 8.80 µg/mL, *P* <.001) and IFX (POCT: 2.39 vs 6.50 µg/mL, *P* <.001; ELISA: 1.70 vs 4.40 µg/mL, *P* <.001). Quartile analysis revealed that higher serum concentrations were associated with maintained response. ROC curve analysis demonstrated good or excellent discrimination for POCT and ELISA in association with LOR (AUC IFX: POCT = 0.82, ELISA = 0.76; AUC ADL: POCT = 0.82, ELISA = 0.81; all *P* <.0001). An overestimation of serum concentrations with POCT was observed.

**Conclusions:**

Serum ADL and IFX POCT concentrations are comparable to ELISA and associated with LOR, indicating its clinical utility.

**Key Messages**

**What is already known?** Point-of-care tests enable rapid measurement of drug concentrations, including anti-TNF agents such as infliximab and adalimumab.

**What is new here?** Infliximab and adalimumab serum concentrations measured by point-of-care tests are associated with loss of response in inflammatory bowel disease patients.

**How can this study help patient care?** These findings implicate the potential of point-of-care tests to provide real-time data, facilitating prompt therapy adjustments to improve patient outcomes.

## Introduction

Inflammatory bowel diseases (IBD)—including Crohn’s disease and ulcerative colitis—are chronic diseases often needing life-long therapy. Among the most frequently used treatments for IBD are anti-tumor necrosis factor (TNF) therapies such as adalimumab (ADL) and infliximab (IFX). Despite their efficacy, up to 30% of patients have primary nonresponse to these treatments and some patients lose their response over time.^1^

Therapeutic drug monitoring (TDM) plays an important role in optimizing treatment for patients experiencing loss of response (LOR).^2,3^ By measuring serum drug concentrations, TDM aids clinicians in tailoring strategies to individual patient needs, thereby improving therapeutic outcomes. However, traditional laboratory-based methods for TDM, such as enzyme-linked immunosorbent assay (ELISA), are associated with significant lag times between sample collection and result availability.^4^ This delay could hold up clinical decision-making and impact patient care.

Point-of-care testing (POCT) offers a promising solution to this challenge by providing rapid measurement of serum drug concentrations at the bedside or in the clinic. Recent studies have validated different POCT techniques in comparison to ELISA using serum as well as whole blood, demonstrating their potential as reliable methods for TDM.^5–7^ The ability of POCT to deliver immediate results facilitates prompt adjustment of dosing.

The clinical utility of POCT for monitoring ADL and IFX serum concentrations in patients with IBD is yet to be demonstrated. Therefore, the aim of this study was to demonstrate the relationship between experiencing LOR and anti-TNF trough levels measured by a POCT compared with reference ELISA.

## Methods

### Study Design

This was a single-center retrospective clinical validation study to test comparability of the POCT and standard ELISA tests and assess its clinical utility, focusing on IFX and ADL serum concentrations. Patients with IBD and available ADL or IFX serum samples at the Amsterdam University Medical Centre were identified. For using the serum on the POCT, patient information forms were sent to all identified patients and written informed consent was obtained. Patients (age ≥ 16 years) with confirmed CD or UC treated with ADL or IFX with at least one available serum sample and a follow-up time of at least 24 months (or less in case LOR occurred sooner) were selected. Patients were excluded if they participated in a clinical trial at the first TDM sample collection during the study period (ie, baseline) or if the serum drug concentration sample date was more than 60 days before having LOR. This time frame was chosen to ensure that the TDM measurement closely reflects the drug exposure at the time of LOR. In the maintained response group, we used the second-to-last serum drug concentration to ensure that LOR did not occur hereafter with this anti-TNF concentration.

### ELISA

The reference serum concentration measurements and anti-drug antibodies (ADA) were performed directly after blood draw at Sanquin Research Institute (Amsterdam, the Netherlands), using a validated in-house bridging ELISA and radioimmunoassay antigen-binding test, respectively. The tests had the following limits: (1) ADL serum level 0.01-250 µg/mL, (2) IFX serum level 0.03-250 µg/mL, (3) ADL ADA level < 12 arbitrary units (AU)/mL-975 AU/mL, and (4) IFX ADA < 12 AU/mL-875 AU/mL.

### POCT

IFX and ADL POCT (ProciseDx Inc., San Diego, CA) was performed on the same serum samples used for the IFX and ADL ELISA tests. The serum samples were stored frozen at –80 °C, thawed at room temperature, and homogenized on a vortex for ~10 seconds. First, 1-mL buffer was dispensed into an IFX cartridge. In addition, 20 µL of the serum sample was dispensed in the cartridge with a pipette, the cartridge was closed, inverted 5 times, and placed in the POCT device to run the test. The POCT device uses time-resolved Fluorescence Resonance Energy Transfer technology to measure the concentrations. The lower and upper limits of the assays were 1.7-77.2 μg/mL for IFX and 1.3-51.5 μg/mL for ADL. Previous studies have validated the ProciseDx POCT system by demonstrating a strong correlation with the standard ELISA method for measuring IFX and ADL levels in blood samples from IBD patients.^7^ This study used blood samples, but our analysis specifically evaluated POCT performance using serum, similar to the ELISA measurements, ensuring direct comparability between the 2 methods.

### Outcomes

The aims of this study were to determine the effect of TDM with IFX or ADL on treatment success and to define optimal serum drug concentration associated with treatment success for both the ELISA and the POCT. Primary outcome was LOR, and this composite endpoint was defined as:

1. Change in therapy (eg, need for addition of corticosteroids, addition immunomodulators, anti-TNF dose increase, addition of a second biologic, or stopping IFX or ADL therapy) as decided by the treating physician.
2. Need for IBD-related surgery (intestinal resection, fistulotomy, stricturoplasty) due to IBD flare, and/or new/recurrent actively draining fistula.
3. Endoscopic deterioration compared with previous endoscopy—both performed during anti-TNF maintenance treatment—defined by a total Simple Endoscopic Score for Crohn Disease (SES-CD) increase of ≥50% or, for ulcerative colitis, an endoscopic Mayo score (eMayo) increase ≥1.

Agreement between ELISA and POCT was assessed using Bland–Altman plots, which enabled us to visualize and quantify the agreement between the 2 methods. These plots illustrate the systematic bias and the dispersion of differences across various concentration ranges. We calculated the mean difference and the limits of agreement (±1.96 standard deviation [SD]) to evaluate how well POCT compared with the reference ELISA method.

### Data Collection

Data was collected by retrospective medical chart review and collected in a structured electronic Case Report Form (Castor EDC, Amsterdam, the Netherlands). Follow-up captured the time from the first available serum sample (baseline) until LOR occurred or until last follow-up at the outpatient clinic during data collection. IFX samples that were not obtained at trough were excluded. The following data were collected: patient and disease characteristics, IBD-related drug and surgical history, dose and interval of IFX, or ADL treatment at baseline. Within 8 weeks surrounding each serum sample collection, data regarding disease activity were collected during clinic and/or day care visit for infusions: clinical (Harvey Bradshaw Index [HBI] for CD, Simple Clinical Colitis Activity Index scores for UC, or if absent Physician Global Assessment), biochemical (C-reactive protein [CRP], fecal calprotectin, serum albumin), and endoscopic (SES-CD or eMayo) data. IFX or ADL concentrations, presence of ADA, and the potential treatment optimization were registered.

### Statistical Analysis

Patients were divided in an LOR and maintained response group for both IFX and ADL. Depending on distribution, descriptive statistics of continuous variables were provided with means and SD or medians and interquartile range (IQR). Categorical variables were described by frequencies and percentages. To investigate differences between the 2 groups at baseline, *P*-values were calculated using a T-test (if normally distributed) or Mann–Whitney U test (if not normally distributed) for continuous variables. To assess the diagnostic accuracy of POCT in detecting LOR, we calculated the positive predictive value (PPV), negative predictive value (NPV), and odds ratio (OR) with corresponding 95% confidence intervals (CIs). To determine the optimal serum drug concentration, a receiver operating characteristic (ROC) graph was plotted. The optimal cutoff was defined by the point with the highest sum of the specificity (spec) and sensitivity (sens) along the ROC curve using the Youden index (J). Quartile analysis was done to assess the percentage of LOR for different cutoffs, and chi-square test was performed to assess differences of LOR between quartiles. The mean difference from all tests (bias) and the upper and lower limit of agreement (95% CI of the bias) are visualized on the Bland–Altman plots. A *P*-value of <.05 was considered statistically significant. All analyses were performed using SPSS (IBM Corp. Released 2019. Version 26.0. Armonk, NY: IBM Corp) or GraphPad Prism (GraphPad Software, Inc. Released 2021, San Diego, CA: GraphPad Software, Inc.).

## Results

**Figure 1.**
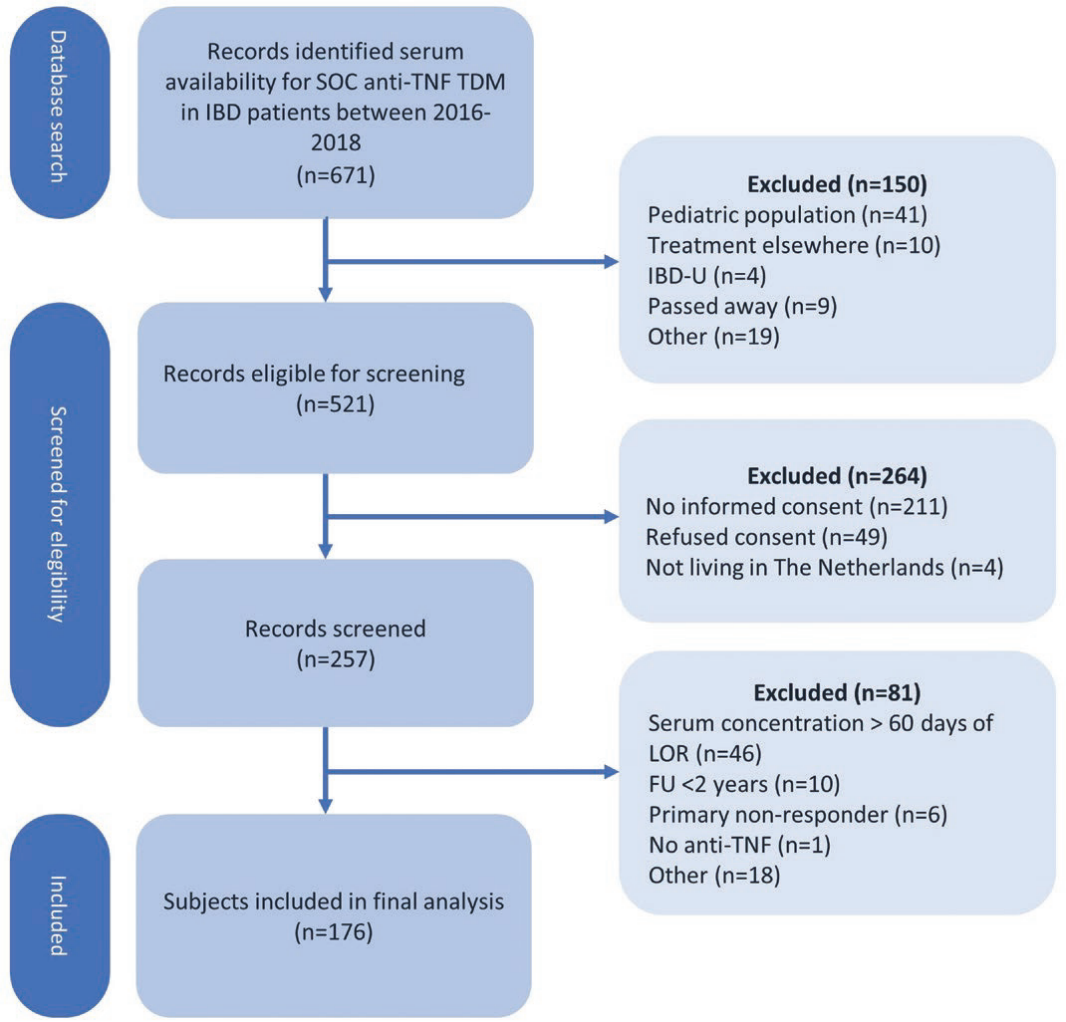
Flow diagram. SOC = Standard of care; TDM = therapeutic drug monitoring; IBD = inflammatory bowel disease; IBD-U = inflammatory bowel disease unspecified; CD = Crohn’s disease; UC = ulcerative colitis; TL = trough level; FU = follow-up; IFX = infliximab; ADL = adalimumab.

A total of 671 records were identified with serum drug concentrations available for standard of care TDM. An opt-in letter was sent to 521 IBD patients to request participation; informed consent was obtained from 257 patients. After screening, 81 patients did not meet the inclusion criteria. Therefore, 176 patients were eventually enrolled in the current study (Figure 1).

Of the analyzed study population, 84/176 (48%) patients were treated with ADL, 92/176 (52%) with IFX (Table 1). The majority of the patients had Crohn’s disease (154/176). Of the patients treated with IFX, 43/92 (47%) patients were treated with a concomitant immunomodulator, and 68/92 (74%) with a standard intravenous dosing scheme (5 mg/kg every 8 weeks). Of the ADL treated patients, 25/84 (30%) were treated with a concomitant immunomodulator and 59/84 (70%) patients were treated with a standard dosing scheme (40 mg every other week).

**Table 1.** Patient and treatment characteristics at baseline. LOR = loss of response. Standard dosing scheme is adalimumab 40 mg every 2 weeks and infliximab 5 mg/kg every 8 weeks. All values are depicted as number (%) or median (IQR) unless stated otherwise.

	Adalimumab			Infliximab		
Patient characteristics	Sustained response (*n* = 47)	LOR (n = 37)	*P*-value	Sustained response (*n* = 37)	LOR (*n* = 55)	*P*-value
Female sex	28 (60%)	18 (49%)	ns	16 (43%)	28 (51%)	ns
Weight	68 (62-75)	75 (60-87)	ns	74 (63-85)	71 (60-84)	ns
Active smoker	8 (20%)	2 (7%)	ns	7 (21%)	9 (21%)	ns
Disease duration at baseline median (min-max)	14 (1-48)	14 (1-52)	ns	13 (1-52)	11 (3-56)	ns
Type of IBD			ns			ns
Crohn’s disease	44 (94%)	32 (87)		29 (78%)	49 (89%)	
Ulcerative colitis	3 (6%)	5 (14%)		8 (22%)	6 (11%)	
**Montreal classification**						
CD age of onset			ns			ns
A1	7 (16)	5 (16%)		4 (13.8%)	14 (29%)	
A2	32 (74%)	20 (65%)		17 (59%)	30 (63%)	
A3	4 (9%)	6 (19%)		8 (28%)	4 (8%)	
CD location			ns			ns
L1	13 (30%)	6 (19%)		8 (28%)	9 (18%)	
L2	11 (25%)	5 (16%)		9 (31%)	19 (39%)	
L3	18 (41%)	18 (56%)		7 (24%)	15 (31%)	
CD behavior			ns			ns
B1	20 (46%)	14 (44%)		16 (55%)	29 (60%)	
B2	17 (39%)	12 (38%)		8 (28%)	11 (23%)	
B3	7 (16%)	6 (19%)		5 (17%)	8 (17%)	
UC extent						
E3	2 (67%)	2 (40%)	ns	6 (75%)	5 (83%)	ns
Previous IBD surgery	20 (43%)	17 (46%)	ns	11 (30%)	20 (36%)	ns
Active perianal disease	4 (9%)	4 (11%)	ns	5 (14%)	2 (4%)	ns
**Treatment characteristics**						
Anti-TNF maintenance therapy duration in months (median, min-max)	36 (1-138)	36 (1-125)	ns	29 (3-149)	34 (1-200)	ns
First biological treatment	20 (43%)	16 (43%)	ns	27 (73%)	41 (75%)	ns
Combination therapy	14 (30%)	11 (30%)	ns	19 (51%)	24 (44%)	ns
Standard dosing scheme	31 (66%)	28 (76%)	ns	26 (70%)	42 (76%)	ns
**Disease activity evaluation at baseline**						
Clinical remission	35 (75%)	21 (57%)	ns	35 (97%)	40 (77%)	.008
CRP (mg/L)	1.4 (0.3-3.1)	3.1 (1.4-13.2)	<.001	1.2 (0.4-3.4)	2.6 (0.7-9.4)	.014
Fecal calprotectin (µg/g)	82 (31-301)	404 (145-1020)	<.001	142 (41-368)	627 (101-1434)	.040
Albumin (g/L)	44 (41-46)	41 (39-45)	.020	43 (41-45)	42 (40-44)	ns
Serum drug concentration (µg/mL)	8.7 (6.3-11.0)	5.3 (2.1-8.8)	<.001	4.4 (2.6-6.5)	2.4 (0.8-4.9)	.003

Median follow-up time was 20 months (IQR 9-38). LOR occurred in a total of 92 patients (37 ADL, 55 IFX). The most frequently reported reasons for LOR in patients treated with ADL and IFX were change of IBD-related medication because of worsening symptoms (14/37 and 22/55, respectively) and elevated inflammatory lab parameters (14/37 and 30/55, respectively). For both patients using ADL and IFX, patients who developed LOR during follow-up had significantly higher CRP and faecal calprotectin levels and lower serum drug concentrations at baseline compared with patients with maintained response. In the sustained remission group, the median time interval between the second-to-last serum drug concentration and the final visit was 5 months (IQR 1-8).

**Figure 2.**
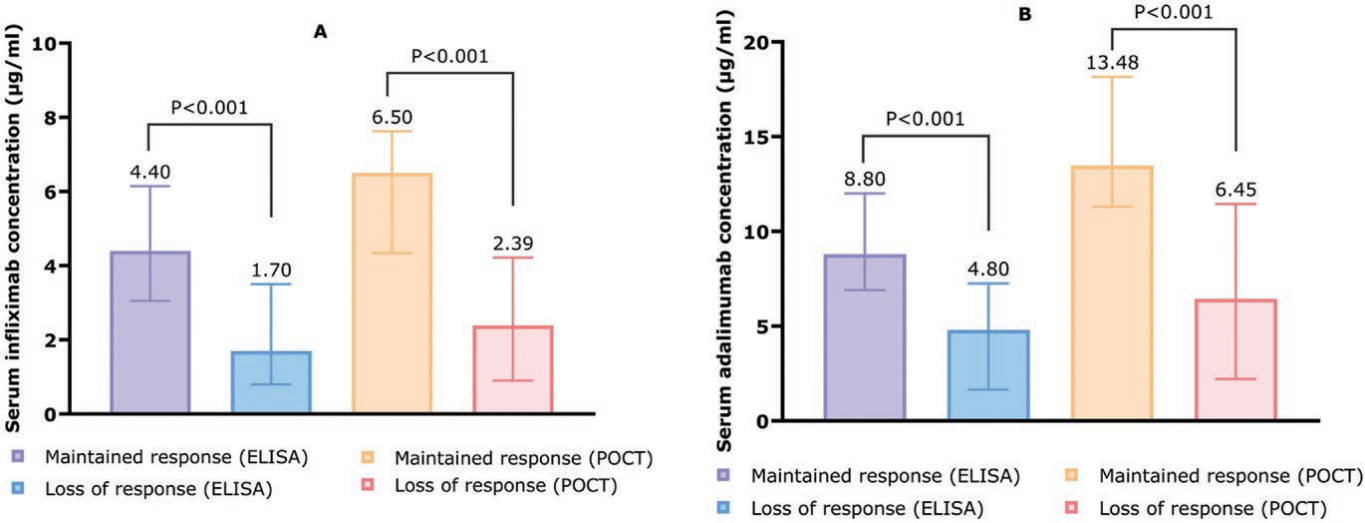
(A) Median IFX concentrations among patients who maintained or lost response, measured with ELISA (left) and POCT (right). (B) Median ADL concentrations among patients who maintained or lost response, measured with ELISA (left) and POCT (right). Concentrations are depicted as µg/mL.

Median serum drug concentrations were significantly lower in patients with LOR compared with those with maintained response, as measured by both techniques for ADL (POCT: 6.45 vs 13.48 µg/mL, *P* <.001; ELISA: 4.80 vs 8.80 µg/mL, *P* <.001) and IFX (POCT: 2.39 vs 6.50 µg/mL, *P* <.001; ELISA: 1.70 vs 4.40 µg/mL, *P* <.001) (Figure 2).

**Figure 3.**
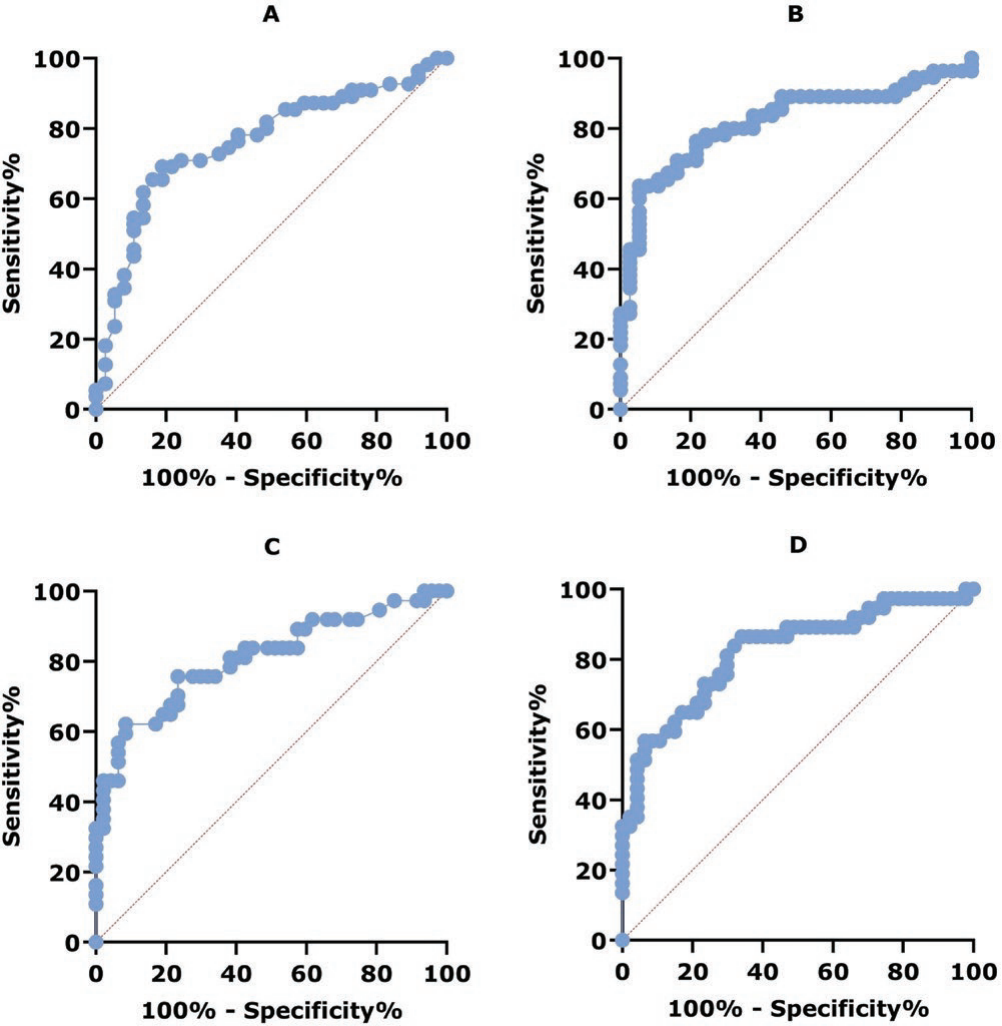
(A) ROC curve for diagnostic performance of IFX serum concentration and LOR measured by ELISA: AUC = 0.76, 95% CI, 0.66-0.86, *P* < .0001. (B) ROC curve for diagnostic performance of IFX serum concentration and LOR measured by POCT: AUC = 0.82, 95% CI, 0.73-0.91, *P* < .0001. (C) ROC curve for diagnostic performance of ADL serum concentration and LOR measured by ELISA: AUC = 0.81, 95% CI, 0.710.91, *P* < .0001. (D) ROC curve for diagnostic performance of ADL serum concentration and LOR measured by POCT: AUC = 0.82, 95% CI, 0.73-0.91, *P* < .0001.

The diagnostic performance of IFX and ADL serum concentrations for LOR was evaluated using ROC curve analysis (Figure 3). The ROC curve for IFX serum concentration measured by ELISA showed an AUC of 0.76 (95% CI, 0.66-0.86), indicating a good level of accuracy (*P* <.0001). The ROC curves for ADL serum concentration measured by the 2 tests and IFX serum concentration measured by POCT showed an AUC of 0.81-0.82, indicating excellent accuracy (*P* <.0001). Optimal cutoff values associated with LOR for ADL were <5.7 µg/mL (sens 62%, spec 92%, PPV 85%, NPV 75%, OR 18 [95% CI, 5.2-59.9]) and <7.3 µg/mL (sens 57%, spec 94%, PPV 88%, NPV 73%, OR 19 [95% CI, 5.1-73.4]) for ELISA and POCT, respectively. For IFX, optimal cutoff values associated with LOR were <2.8 µg/mL (sens 69%, spec 81%, PPV 84%, NPV 64%, OR 9.5 [95% CI, 3.5-26.1]) and <2.7 µg/mL (sens 64%, spec 95%) for ELISA and POCT, respectively.

**Figure 4.**
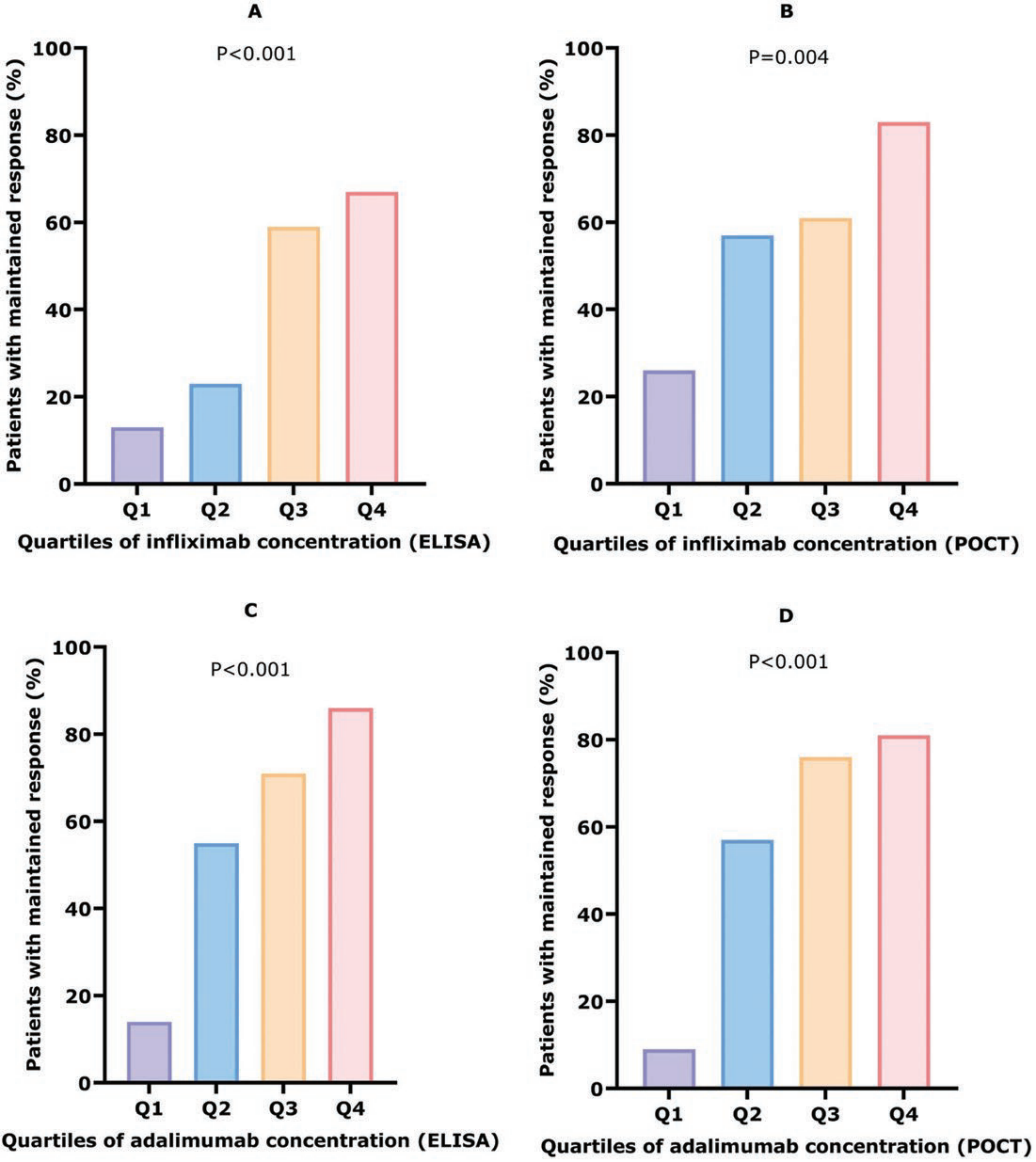
Serum drug concentration quartile analysis for maintained response. (A) Serum infliximab concentrations measured by ELISA. Q1: ≤ 1.3 (*N* = 3/24), Q2: 1.4-2.8 (*N* = 5/22), Q3: 2.9-4.6 (*N* = 13/22), Q4: > 4.7 (*N* = 16-24), *P* < .001. (B) Serum infliximab concentrations measured by POCT. Q1: ≤ 3.4 (*N* = 6/23), Q2: 3.5-6.5 (*N* = 13/23), Q3: 6.6-11.9 (14/23), Q4: > 11.9 (*N* = 19/23), *P* = .004. (C) Serum adalimumab concentrations measured by ELISA. Q1: ≤ 4.8 (*N* = 3/22), Q2: 4.9-7.3 (*N* = 11/20), Q3: 7.3-9.7 (*N* = 15/21), Q4: > 9.7 (*N* = 18/21), *P* < .001. (D) Serum adalimumab concentrations measured by POCT. Q1: ≤ 6.6 (*N* = 2/21), Q2: 6.6-11.5 (*N* = 12/21), Q3: 11.5-15.0 (*N* = 16/21), Q4: > 15.0 (*N* = 17/21), *P* < .001.

Quartile analysis shows a clear dose–response relationship for IFX and ADL, measured by both ELISA and POCT (Figure 4). For IFX measured by ELISA, a significant difference in maintained response was observed across quartiles (Q1: ≤1.3, Q2: 1.4-2.8, Q3: 2.9-4.6, Q4: >4.7 µg/mL; *P* <.001). Similarly, IFX measured by POCT showed significant differences in sustained response across quartiles (Q1: ≤3.4, Q2: 3.5-6.5, Q3: 6.6-11.9, Q4: >11.9 µg/mL; *P* =.004). For ADL, ELISA revealed significant differences in sustained response (Q1: ≤4.8, Q2: 4.9-7.3, Q3: 7.4-9.7, Q4: >9.7 µg/mL; *P* <.001). The POCT measurements for ADL also indicated significant variations in sustained response across quartiles (Q1: ≤6.6, Q2: 6.6-11.5, Q3: 11.5-15.0, Q4: >15.0 µg/mL; *P* <.001).

**Figure 5.**
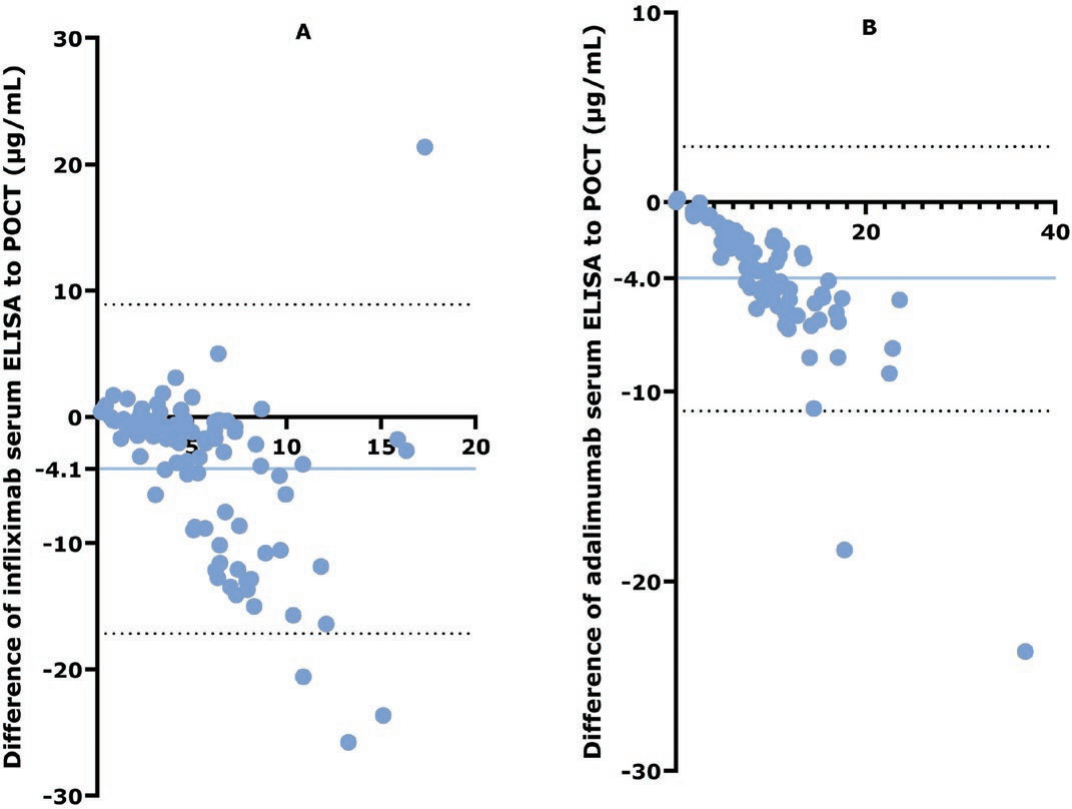
Bland–Altman plots. (A) Comparison of ELISA with IFX POCT. (B) Comparison of ELISA with ADL POCT. The *y*-axis shows the differences between each test result and the *x*-axis shows the mean of each test result. The blue horizontal line represents the bias (the mean difference between ELISA and POCT), with the absolute number of the bias depicted on the *y*-axis. Dashed black horizontal lines represent the upper and lower limits of the bias. Blue dots represent each individual participant.

Bland–Altman plots showed a negative bias for both IFX and ADL ELISA compared with POCT, indicating that POCT systematically overestimated anti-TNF concentrations (Figure 5). The systematic overestimation and increased dispersion at higher concentrations indicate that while POCT can be used, it may not fully align with ELISA at higher concentration levels.

ADA against IFX, measured by radioimmunoassay antigen-binding test, were present in 3/92 patients, all having LOR. Median ADA against IFX levels were 45 AE/mL (IQR 18-880). In these patients, all serum concentrations were below the lower detection limit of the used assay for both ELISA and POCT. ADA against ADL were present in 14/84 patients, 11/14 had LOR, and median ADA levels against ADL were 23 AE/mL (IQR 15-980). In these patients, median ADL serum concentrations were 3.4 µg/mL (IQR 0.01-6.9) measured by ELISA and 4.8 µg/mL (IQR 0.01-9.9) measured by POCT.

## Discussion

The objectives of this study were to evaluate the association between LOR in IBD patients and IFX or ADL serum concentrations measured using a POCT compared with ELISA. Our findings indicate that POCT measurements of IFX and ADL are closely associated with LOR and are comparable to those obtained through traditional reference methods.

Our results demonstrated that drug concentrations were significantly lower in patients with LOR compared with those with sustained response for both IFX and ADL, measured by both POCT and ELISA. This is in line with previous research indicating that lower drug concentrations are associated with reduced therapeutic efficacy.^8^ The ROC curve analysis further supported the reliability of POCT in predicting LOR, with AUC demonstrating high accuracy. These findings suggest that POCT is a reliable tool for monitoring drug concentrations, potentially enabling timely adjustment in therapy.

POCT also offers the advantage of real-time and home-based monitoring, allowing patients to have their drug levels assessed outside of traditional clinical settings. This could facilitate dose adjustments in real-time based on home sampling. Clinicians would then have sufficient time to interpret the POCT drug concentration and make dose adjustments prior to the next infusion visit or subcutaneous administration. This approach would enhance the clinical utility of proactive TDM and may optimize treatment efficacy.^9,10^

Moreover, with the expectation that an increasing number of Crohn’s disease patients will be treated with a top-down approach with anti-TNF therapies, timely TDM becomes even more crucial.^11^ While our study utilized stored serum samples for evaluation, recent research has shown that POCT correlates well with finger prick capillary blood sampling, making it feasible for patients to perform sampling at home.^7^ By eliminating the need for venipuncture and providing rapid results, POCT can streamline the TDM process.^5,12^

Immunogenicity assessment revealed the presence of ADA against IFX and ADL, as determined by radioimmunoassay antigen-binding test. Among the 92 patients included in the study, only 3 exhibited ADA against IFX, all of whom experienced LOR, with all serum concentrations falling below the lower limit of detection for the assay employed, regardless of whether measured by ELISA or POCT. For patients treated with ADL, ADA were detected in 14 out of 84 patients, with the majority of these patients experiencing LOR. Interestingly, median serum concentrations of ADL were often measurable and comparable between ELISA and POCT, highlighting the consistency in assessing drug concentrations. However, due to the limitations of the POCT device in detecting ADA, assessment of immunogenicity remained dependent on the reference lab method. Recent advances have led to the development of POCT platforms capable of detecting ADA, offering the potential for comprehensive and prompt TDM.^6^

POCT drug levels were generally higher compared with ELISA, indicating that different cutoff values may be necessary for accurate assessment with POCT. For IFX, however, the optimal cutoff values for detecting LOR were comparable (<2.8 µg/mL for ELISA and <2.7 µg/mL for POCT). In contrast, for ADL, optimal cutoff values were <5.7 µg/mL for ELISA and <7.3 µg/mL for POCT. These findings suggest that while POCT is generally accurate, adjustments in cutoff values are necessary for different drugs to ensure reliable detection of LOR, particularly for ADL. These differences also highlight the need for harmonization across assay platforms, to support consistent interpretation of drug concentrations and facilitate broader implementation of TDM in clinical practice.

The strength of our study is the inclusion of a sizeable cohort of IBD patients and the careful definition of our outcome parameters. Limitations include the retrospective nature of data collection, which also prevented us from acting upon the TDM measurements in real clinical practice. Moreover, the POCT serum concentrations were slightly different compared with the reference concentrations, in line with previous publications on the same POCT device.^5^ However, despite the slightly increased drug concentrations, this did not affect the clinical validity because this was comparable in all different groups. Another limitation is the higher error rate observed in POCT at higher serum drug concentrations. This is particularly relevant in clinical scenarios where higher serum concentrations are desired, such as in patients with fistulising Crohn’s disease or during induction therapy.^4^ In these situations, achieving and accurately measuring higher drug concentrations is crucial for effective treatment. As our study primarily included patients on maintenance therapy, further studies are needed to confirm whether POCT performs reliably in the higher concentration ranges typically observed during induction. Prospective and randomized controlled trials are warranted to evaluate the clinical utility and cost-effectiveness of POCT-guided treatment, particularly during induction therapy.

In conclusion, POCT for IFX and ADL drug concentrations is a promising tool for managing IBD, providing comparable accuracy to traditional methods and enabling therapeutic adjustments in a prompt manner. Incorporating POCT into clinical practice could enhance patient management by minimizing LOR and timely optimization of therapy.

## Data Availability

The data underlying this article cannot be shared publicly due to the privacy of individuals that participated in the study. Pseudo-anonymised data will be shared on reasonable request to the corresponding author.

## References

[1] Wong U, Cross RK. Primary and secondary nonresponse to infliximab: Mechanisms and countermeasures. Expert Opin Drug Metab Toxicol. 2017;13(10):1039-1046. doi: https://doi.org/10.1080/17425255.2017.1377180 28876147

[2] Irving PM, Gecse KB. Optimizing therapies using therapeutic drug monitoring: current strategies and future perspectives. Gastroenterology. 2022;162(5):1512-1524. doi: https://doi.org/10.1053/j.gastro.2022.02.014 35167865

[3] Feuerstein JD, Nguyen GC, Kupfer SS, Falck-Ytter Y, Singh S; American Gastroenterological Association Institute Clinical Guidelines Committee. American gastroenterological association institute guideline on therapeutic drug monitoring in inflammatory bowel disease. Gastroenterology. 2017;153(3):827-834. doi: https://doi.org/10.1053/j.gastro.2017.07.032 28780013

[4] Papamichael K, Afif W, Drobne D, et al; International Consortium for Therapeutic Drug Monitoring. Therapeutic drug monitoring of biologics in inflammatory bowel disease: Unmet needs and future perspectives. Lancet Gastroenterol Hepatol. 2022;7(2):171-185. doi: https://doi.org/10.1016/S2468-1253(21)00223-5 35026171 PMC10187071

[5] Volkers A, Löwenberg M, Braad M, et al Validation study of novel point-of-care tests for infliximab, adalimumab and c-reactive protein in capillary blood and calprotectin in faeces in an ambulatory inflammatory bowel disease care setting. Diagnostics (Basel). 2023;13(10):1712. doi: https://doi.org/10.3390/diagnostics13101712 37238198 PMC10217227

[6] Valdés-Delgado T, Aguado-Paredes A, Merino-Bohórquez V, et al Performance of a new rapid point-of-care test for infliximab levels in patients with inflammatory bowel disease: a comparison to elisa. Dig Dis Sci. 2024;69(1):228-234. doi: https://doi.org/10.1007/s10620-023-08139-1 37943382 PMC10787688

[7] Bonazzi E, Maniero D, Lorenzon G, et al Comparing point-of-care technology to elisa testing for infliximab and adalimumab levels in adult inflammatory bowel disease patients: a prospective pilot study. Diagnostics (Basel). 2024;14(19):2140. doi: https://doi.org/10.3390/diagnostics14192140 39410544 PMC11482612

[8] Kennedy NA, Heap GA, Green HD, et al; UK Inflammatory Bowel Disease Pharmacogenetics Study Group. Predictors of anti-tnf treatment failure in anti-tnf-naive patients with active luminal crohn’s disease: a prospective, multicentre, cohort study. Lancet Gastroenterol Hepatol. 2019;4(5):341-353. doi: https://doi.org/10.1016/S2468-1253(19)30012-3 30824404

[9] Bossuyt P, Pouillon L, Claeys S, et al Ultra-proactive therapeutic drug monitoring of infliximab based on point of care testing in inflammatory bowel disease: Results of a pragmatic trial. J Crohns Colitis. 2022;16(2):199-206. doi: https://doi.org/10.1093/ecco-jcc/jjab127 34297099

[10] Syversen SW, Jørgensen KK, Goll GL, et al Effect of therapeutic drug monitoring vs standard therapy during maintenance infliximab therapy on disease control in patients with immune-mediated inflammatory diseases: a randomized clinical trial. JAMA. 2021;326(23):2375-2384. doi: https://doi.org/10.1001/jama.2021.21316 34932077 PMC8693274

[11] Noor NM, Lee JC, Bond S, et al; PROFILE Study Group. A biomarker-stratified comparison of top-down versus accelerated step-up treatment strategies for patients with newly diagnosed crohn’s disease (profile): a multicentre, open-label randomised controlled trial. Lancet Gastroenterol Hepatol. 2024;9(5):415-427. doi: https://doi.org/10.1016/S2468-1253(24)00034-7 38402895 PMC11001594

[12] Otten AT, van der Meulen HH, Steenhuis M, et al Clinical validation of a capillary blood home-based self-sampling technique for monitoring of infliximab, vedolizumab, and c-reactive protein concentrations in patients with inflammatory bowel disease. Inflamm Bowel Dis. 2024;30(3):325-335. doi: https://doi.org/10.1093/ibd/izad103 37265165 PMC10906358

